# Combining XCO_2_ Measurements Derived from SCIAMACHY and GOSAT for Potentially Generating Global CO_2_ Maps with High Spatiotemporal Resolution

**DOI:** 10.1371/journal.pone.0105050

**Published:** 2014-08-13

**Authors:** Tianxing Wang, Jiancheng Shi, Yingying Jing, Tianjie Zhao, Dabin Ji, Chuan Xiong

**Affiliations:** State Key Laboratory of Remote Sensing Science, Institute of Remote Sensing and Digital Earth, Chinese Academy of Sciences. Beijing, China; University of Oxford, United Kingdom

## Abstract

Global warming induced by atmospheric CO_2_ has attracted increasing attention of researchers all over the world. Although space-based technology provides the ability to map atmospheric CO_2_ globally, the number of valid CO_2_ measurements is generally limited for certain instruments owing to the presence of clouds, which in turn constrain the studies of global CO_2_ sources and sinks. Thus, it is a potentially promising work to combine the currently available CO_2_ measurements. In this study, a strategy for fusing SCIAMACHY and GOSAT CO_2_ measurements is proposed by fully considering the CO_2_ global bias, averaging kernel, and spatiotemporal variations as well as the CO_2_ retrieval errors. Based on this method, a global CO_2_ map with certain UTC time can also be generated by employing the pattern of the CO_2_ daily cycle reflected by Carbon Tracker (CT) data. The results reveal that relative to GOSAT, the global spatial coverage of the combined CO_2_ map increased by 41.3% and 47.7% on a daily and monthly scale, respectively, and even higher when compared with that relative to SCIAMACHY. The findings in this paper prove the effectiveness of the combination method in supporting the generation of global full-coverage XCO_2_ maps with higher temporal and spatial sampling by jointly using these two space-based XCO_2_ datasets.

## Introduction

In recent years, global warming caused by emission of CO_2_ has attracted considerable attention from the public. During the past decade, although tremendous efforts have been made toward improving the understandings of the mechanism between CO_2_ increase in the atmosphere and global warming, some uncertainties still exist in the spatiotemporal characteristics of CO_2_ sinks/sources on regional and global scales due to the lack of high-density measurements of such variables with good accuracy [1], [2]. To date, the estimates of CO_2_ flux from inverse methods rely mainly on ground-based measurements [3], [4]. Although providing highly accurate atmospheric CO_2_ records, the traditional ground-based networks intrinsically suffer from sparse spatial coverage [2], [5]. Satellite-based measurements with various spatial and temporal resolutions provide a unique opportunity to accurately map atmospheric CO_2_ in both daytime and nighttime over large areas, thus having the potential to bridge this gap. As a result, various satellite-based platforms have been equipped in recent years for deriving the CO_2_ concentrations.

Generally, methods for retrieving CO_2_ from space can be grouped into two categories: (1) inferring CO_2_ concentrations by measuring shortwave infrared (SWIR) reflected solar radiation around 1.6 and 2.0 µm with sufficient spectral resolution. This includes the Greenhouse gases Observing SATellite (GOSAT), operating since 2009 [6], the Scanning Imaging Absorption spectrometer for Atmospheric CartograpHY (SCIAMACHY), in orbit since 2002 [7], and the second Orbiting Carbon Observatory (OCO-2), which, as a rebuild of OCO [8], [9], is planned to be launched in July 2014. In addition, CarbonSat will also be scheduled to be launched in 2018 (http://www.iup.uni-bremen.de/carbonsat/). These measurements have a nearly uniform sensitivity to CO_2_ from the surface up through the middle troposphere, and thus are frequently used to derive the column-average dry air mole fraction of atmosphere CO_2_ (XCO_2_) during the daytime; (2) retrieving CO_2_ concentrations by interpreting the recorded spectra of the Earth-atmosphere system in thermal infrared (TIR) bands (around 15 µm). Instruments that work in such a way include AIRS [10], [11], IASI [12], [13], and FTS (Band 4) of GOSAT [6]. These measurements bring the advantage that they can detect CO_2_ during both day and night time, while the lack of sensitivity in the lower troposphere makes them inappropriate to estimate CO_2_ near the surface where the largest signals of CO_2_ sources and sinks occur [1]. The complementarities of these platforms allow us to combine the SWIR and TIR measurements for obtaining enhanced understanding of CO_2_ spatiotemporal variations globally. Since XCO_2_ is much less affected by vertical transport of CO_2_, it is particularly useful for investigation of CO_2_ sources and sinks using inversion modeling [14], [15]. On the other hand, the spatial and temporal variations in XCO_2_ are even smaller than that in the surface CO_2_; therefore, unprecedented measurement precision and accuracy are highly required for such column measurements [16]–[19]. SCIAMACHY (operation stopped in April 2012) and GOSAT are two typical instruments that can be used to derive XCO_2_ from space, and a variety of retrieval algorithms have been developed for SCIAMACHY [1], [20]–[27] and GOSAT [2], [4], [5], [28]–[30] with eyes on improving XCO_2_ retrieval accuracy to a great extent. At present, a number of XCO_2_ products have been released. These will definitely enhance our understanding of the global carbon cycle.

**Figure 1:**
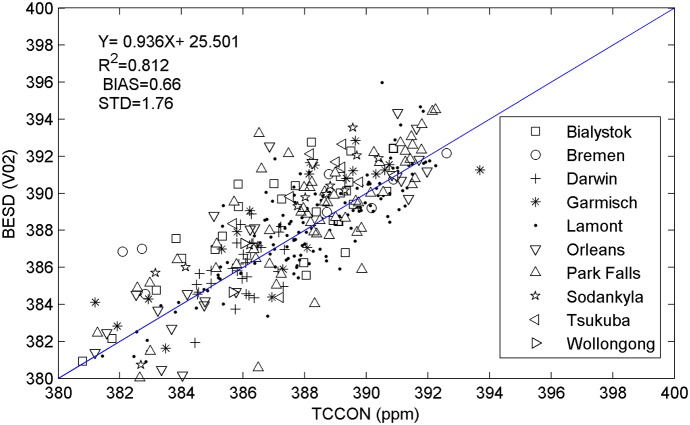
Validation of the BESD products against *in situ* TCCON CO_2_ measurements over globe for 2009–2010.

Unfortunately, almost all typical instruments currently used to derive atmospheric CO_2_ concentration are working in the infrared spectral range (less than 16 µm). Thus, except for the instrument’s observation mode (for example, GOSAT observes in lattice points), the spatial coverage of the derived CO_2_ is severely restricted by the presence of clouds. In addition, the lower signal-to-noise level over ice/snow covered surfaces and ocean for SWIR instruments (e.g., SCIAMACHY) also contributes to the CO_2_ sparse coverage. For instance, it has been pointed out that only about 10% of GOSAT data can be used for retrieval of XCO_2_ due to the cloud contaminations [4]. The amount of CO_2_ measurements will be even smaller if additional screening criteria such as quality of spectral fit, aerosol loadings, etc. are further applied. Although the amount of remaining CO_2_ measurements from certain space-based instruments may largely surpass that of ground-based sites, it is still not sufficient enough for accurately quantifying the spatiotemporal distribution of CO_2_ over the global scale. As a result, it is greatly desired to jointly use these available CO_2_ measurements derived from various space-based data. Recently, a novel method has been proposed for combining CO_2_ values from seven different algorithms, and a new Level-2 CO_2_ database (EMMA) from one algorithm is composed according to the median of monthly average of seven CO_2_ products in each 10°×10° latitude/longitude grid box [31]. In fact, this method cannot increase the number of CO_2_ observations but chooses a product with moderate oscillation among the available products. Despite the usefulness of the XCO_2_ measurements (Level 2) in their own right, further spatiotemporal analysis for interpreting their scientific merit is essentially necessary due to the retrieval uncertainties and sparse coverage of such Level-2 observations [32]. For this point, many works have attempted to generate global full-coverage (i.e., Level 3) maps from XCO_2_ values derived from single satellite observations using a geospatial statistics approach [32]–[34]. However, as reflected in these studies (for instance, Fig. 1 in the work of [33]), a compromise has to be made between the interpolated accuracy and the spatiotemporal resolution of Level-3 product because of the limited amount of Level-2 XCO_2_ observations being used. For this point, instead of using Level-2 XCO_2_ from a single dataset (e.g., GOSAT or OCO-2) as performed in the existing literature, we attempt to explore the potential of combining two CO_2_ datasets (GOSAT and SCIAMACHY) in assisting in global Level-3 generation, aiming to: (1) propose a general strategy for combining (fusing) various CO_2_ datasets with different instruments, algorithms, averaging kernels, etc.; and 2) increase the number of daily CO_2_ points (utilized in Level-3 map interpolations) through the combination of two datasets, so that potentially improved Level-3 maps with higher accuracy and shorter time scale can be generated. The better the interpretation of the satellite-based CO_2_ observations one can make, the higher the resolution (both temporal and spatial) of the generated global CO_2_ maps.

## Datasets

For GOSAT, the Fourier transform spectrometer (FTS) on GOSAT is the fundamental unit to retrieve atmospheric CO_2_ and CH_4_. It observes sunlight reflected from the earth’s surface, and light emitted from the atmosphere and the surface. It is composed of three narrow bands in the SWIR region (0.76, 1.6, and 2.0 µm) and a wide TIR band (5.5–14.3 µm) at a spectral and spatial resolution of 0.2 cm^−1^ and 10.5 km, respectively [35]. Specifically, four CO_2_ products from GOSAT have currently been released to the public: University of Leicester product [9], [36], the RemoTeC product [28], NIES GOSAT product [35] and the product generated by NASA’s Atmospheric CO_2_ Observations from Space (ACOS) team (hereafter called ACOS product) [2], [30]. The difference between some of the above mentioned products with various versions have been investigated in a recent study [37]. In the present paper, the ACOS product of 2009–2010 with version v2.9 has been employed.

SCIAMACHY was successfully launched on board Environmental Satellite (ENVISAT) in 2002 (unfortunately ceased in April 2012), which is a detector elements satellite spectrometer covering the spectral range 0.24–2.38 µm with a moderate spectral resolution of about 0.2–1.6 nm, and spatial resolution at nadir of 60×30 km [7]. It has eight spectral channels, with 1024 individual detector diodes for each band, observing the spectral regions 0.24–1.75 µm (band 1–6), 1.94–2.04 µm (band 7), and 2.26–2.38 µm (band 8) simultaneously in nadir and limb and solar and lunar occultation viewing geometries [22]. As mentioned in Section 1, till today, a number of CO_2_-retrieval algorithms have been developed for SCIAMACHY. The IUP/IFE of University of Bremen has released two XCO_2_ products, i.e., WFM-DOAS product [21], [22] and the Bremen Optimal Estimation DOAS (BESD) product [1], [26]. In this study, the BESD product with the versions of v02.00.08 for 2009–2010 is used.

In addition, CO_2_ profiles of CT [38] are also collected here to allow the data mentioned above to be properly fused. CT is a NOAA data assimilation system, which provides the 3D profiles of CO_2_ mole fractions in the atmosphere over the globe. For this study, CT data with version CT2011 is collected. This dataset provides global CO_2_ profiles with 3°×2° latitude/longitude grid and 3 hours temporal resolution (a total 8 times from 01 to 22 in UTC) spanning the time period from January 2000 to December 2010. The CT dataset is used here mainly to assist in adjusting and time-shifting of the two CO_2_ products being combined.

## Methodologies

For combining the different space-based CO_2_ measurements, three steps are adapted in this study. First, taking the global ground measurements of CO_2_ as reference, remove the bias of the individual CO_2_ retrievals for ensuring the accuracy of the fused CO_2_ product; then make some adjustment for both the ACOS and BESD products, so that they can be physically comparable and thus combined; finally fuse the ACOS and BESD CO_2_ products considering their retrieval uncertainties, spatial scales, differences in averaging kernels and overpass times, etc.

### 3.1 Global bias corrections

Removal of any global bias of the retrieved CO_2_ when compared with the ground *in situ* measurements is essential before performing joint use. Many researches [4], [25] frequently pointed out that CO_2_ retrievals from GOSAT are low biased with different levels due to the uncertainties in pressure, radiometric calibration, line shape model, cloud and aerosol scattering, etc. Fortunately, a recent study has proposed a method for evaluating systematic errors in CO_2_ and showed that the new version of ACOS product (v2.9) has a low global bias (<0.5 ppm) [39]. Thus, there is no global bias correction for the ACOS product being conducted here, but only the ACOS retrievals that pass the filter of table B1 in the work of [38] and marked as “good” in the quality flag are used. For the BESD product, we select Total Carbon Column Observing Network (TCCON) [15] measurements for 2009–2010 as the ground truth to determine its global bias. Specifically, BESD retrievals within ±2.5° and ±2.5° latitude/longitude box centered at each TCCON site and the mean FTS value (within ±1 h time window of satellite overpass time) are extracted and compared (totally ten TCCON sites are utilized). The coincidence criteria mentioned above ultimately yield a total of 338 pairs of CO_2_ measurements. The comparison result is shown in Fig. 1.

### 3.2 Retrieval adjustments

As pointed out by most researchers, it is not reasonable to directly compare or use two XCO_2_ measurements. A suitable way to do that is to take the a priori profiles and variations in averaging kernel into account during the comparison [26], [40]. To tackle the a priori issue, after correcting their global biases, both BESD and ACOS products are adjusted for a common a priori profile, which we assume to be the CT profile interpolated at the middle of the two overpass times (Equation (1)). Specifically, the a priori CO_2_ profile of both the ACOS and BESD are first interpolated or extrapolated to the level of the CT CO_2_ profile according to their pressure layers. After interpolation, the a priori profiles for both ACOS and BESD have the same dimension as the CT profile. Here the reason we take the CT profile at the middle of the two overpass times is that the time difference for GOSAT (1∶00 pm) and SCIAMACHY (10∶00 am) is relative large (3 hours), if we take one satellite time as reference, the induced error would be large for the other satellite measurements considering the CO_2_ natural diurnal variation. So a middle time between these two satellite overpass times is selected for minimizing the CO_2_ uncertainties during the adjustment.
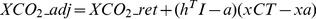
(1)Here, 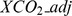 is the adjusted XCO_2_ for ACOS or BESD; 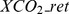 corresponds to retrieved XCO_2_ of ACOS or BESD; *a* is the column-averaging kernel (row vector) of ACOS or BESD; *h* is pressure-weighting function (column vector); *I* is an identity matrix; 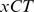 and 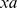 (column vectors) are the common CT CO_2_ profile and the corresponding a priori CO_2_ profile for ACOS or BESD, respectively.

While it is not trivial to accurately consider the smoothing error without an estimate of the true atmospheric variability which is generally not readily available for most cases [39]. Fortunately, some works revealed that the smoothing error is generally small [26], [39]. Consequently, for the remainder of this paper, only the adjustment in Equation (1) is applied for both the ACOS and BESD CO_2_ products (after bias corrections).

### 3.3 Combination and time shifting

Based on the processes described above, the world is divided into a number of 0.5°×0.5°latitude/longitude grid box (totally 720×360). For each grid cell, Equation (2) is used to combine the corresponding CO_2_ measurements within that grid.
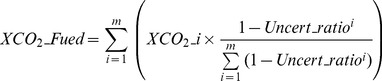
(2)where 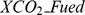 is the combined XCO_2_; *m* is the total number of space-based CO_2_ retrievals (ACOS and/or BESD) within a certain grid; 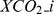 is the *i*th XCO_2_ retrieval in a grid for which the global bias and Equation (1) are supposed to be applied; 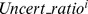 is the ratio of uncertainty of the *i*th XCO_2_ retrieval to its XCO_2_ value.

Please note that since different CO_2_ retrievals have distinct overpass times, it is necessary to unify them to avoid uncertainties induced from the time discrepancy before fusion. To this end, a method for considering the CO_2_ shifting along time has been developed (Equation (3)). First, designate a specific time or select one overpass time as reference, then transfer CO_2_ measurements at various overpass times to that of the reference time by interpolating the CT CO_2_ at temporal scale. Here, it should be pointed out that despite the CO_2_ absolute values of CT not being accurate enough, the daily cycle pattern of atmospheric CO_2_ it reflects is assumed to be correct.
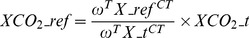
(3)Here, 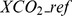 is the transformed XCO_2_ (ACOS or BESD) at the reference time; 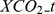 is the retrieved XCO_2_ from ACOS or BESD at overpass time *t*; 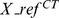 and 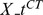 are CO_2_ profiles of CT at times of reference and *t*, respectively; 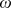 is the pressure-weighting vector (column vector).

Based on the time-shifting strategy proposed here, a global CO_2_ map at any specific time can be theoretically produced by employing the pattern of the CO_2_ daily cycle reflected by CT data. For instance, we can unify all XCO_2_ retrievals being combined with various overpass times to that of UTC = 1.

## Results

Evaluation analysis showed that the global bias for the BESD product is generally small. In this study, the bias of the BESD product is corrected by subtracting 0.6 ppm from all XCO_2_ values according to the results in Fig. 1. Although the systematic bias of the XCO_2_ retrievals is removed, it is supposed that the error characteristics (random error) within the data are still unchanged. The bias-corrected XCO_2_ retrievals of both ACOS and BEDS are used as fundamental data for the combination algorithm.

**Figure 2:**
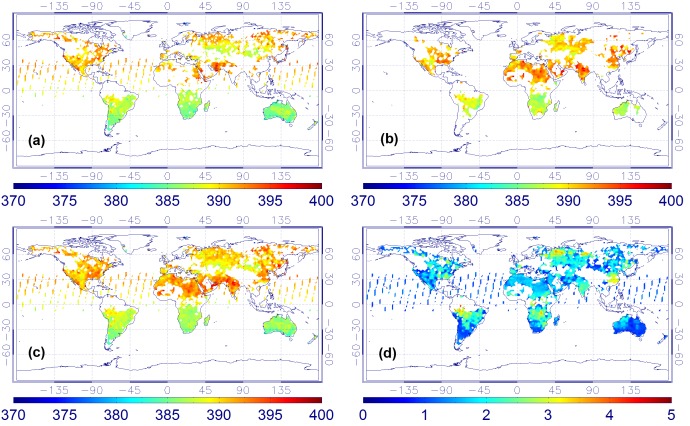
XCO_2_ monthly mean maps in May of 2010. ((a) ACOS XCO_2_, (b) BESD XCO_2_, (c) combined product, and (d) XCO_2_ uncertainties of the combined product).

By applying the series of processes shown in Section 3, daily, weekly, as well as monthly maps of combined XCO_2_ for 2009 and 2010 are generated. Here, as an example, only four maps (from May to August) of monthly mean XCO_2_ of 2010 are shown here (Fig. 2–Fig. 5). In addition, the total XCO_2_ uncertainties of the combined product which mainly depend on the uncertainties of the original ACOS or BESD XCO_2_ retrievals are also illustrated.

**Figure 3:**
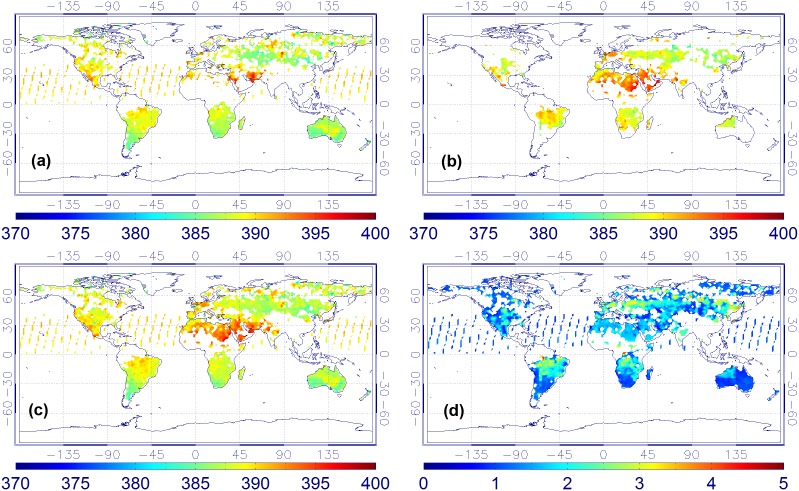
XCO_2_ monthly mean maps in June of 2010. ((a) ACOS XCO_2_, (b) BESD XCO_2_, (c) combined product, and (d) XCO_2_ uncertainties of the combined product).

**Figure 4:**
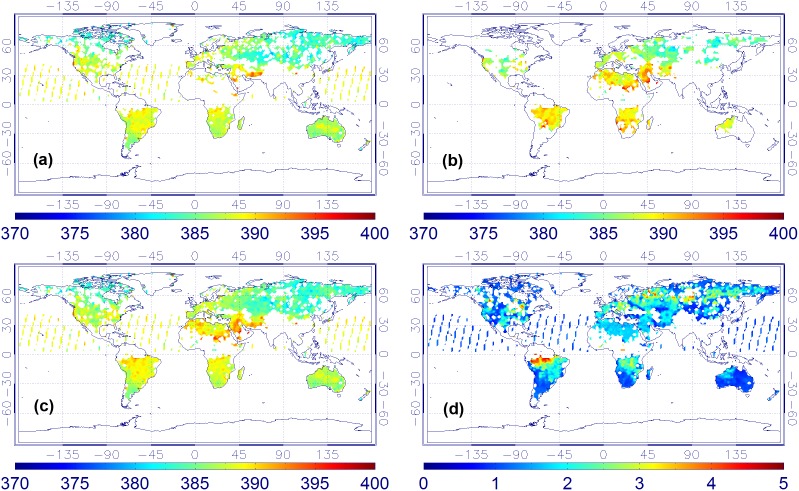
XCO_2_ monthly mean maps in July of 2010. ((a) ACOS XCO_2_, (b) BESD XCO_2_, (c) combined product, and (d) XCO_2_ uncertainties of the combined product).

**Figure 5:**
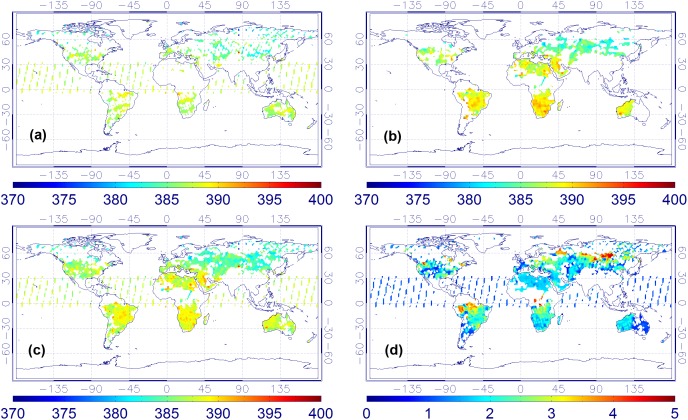
XCO_2_ monthly mean maps in August of 2010. ((a) ACOS XCO_2_, (b) BESD XCO_2_, (c) combined product, and (d) XCO_2_ uncertainties of the combined product).

**Figure 6:**
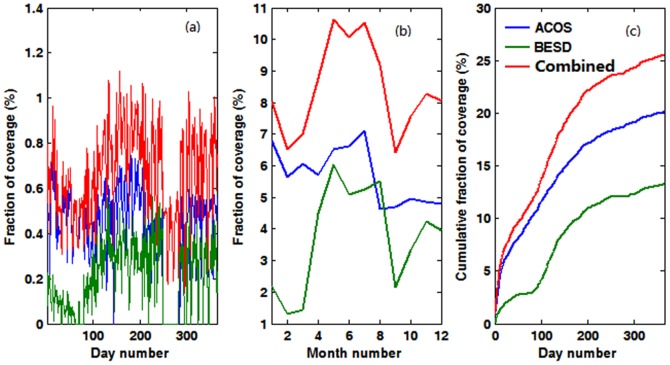
XCO_2_ fraction of coverage of ACOS, BESD, and combined products. (a) Daily coverage. (b) Monthly coverage. (c) Cumulative coverage.

From Fig. 2–Fig. 5, it is not difficult to observe that the combined data realize the physical complementary of the two products in terms of spatial coverage. The number of valid CO_2_ measurements in the fused product is the union of the CO_2_ data from both the ACOS and BESD at the same geographical location. In addition, the combined XCO_2_ demonstrates similar spatiotemporal characteristics with that of ACOS and BESD over the globe, which implies that all processes associated with the combination do not distort the essential information of the original XCO_2_ products (ACOS or BESD). Similar findings can also be observed in the daily mean and weekly mean XCO_2_ maps. To quantitatively investigate the improvement of fused XCO_2_ in spatial coverage, the fractional coverage of all three variables (ACOS, BESD, and combined XCO_2_) on both daily and monthly scales is calculated (Fig. 6). From Fig. 6, it can be seen that the average global coverage of ACOS and BESD is around 0.46% and 0.21%, respectively, on a daily scale. The monthly mean coverage of such products accounts for about 5.70% and 3.75%, respectively. While spatial coverage of combined XCO_2_ can reach up to 0.65% and 8.42% on daily and monthly scales, respectively, it accounts for increments of 41.3% and 47.7% on the daily and monthly scales with respect to that of GOSAT and it is even higher relative to the coverage of SCIAMACHY. Likewise, the cumulative fraction of coverage of the combined XCO_2_ has risen to 25% when compared with 20% and 13% for ACOS and BESD, respectively. The increase in the XCO_2_ spatial coverage indicates the potential advantage of the combined XCO_2_ observations in generating global Level-3 XCO_2_ maps when compared with any single dataset by providing more satellite-based XCO_2_ retrievals used for optimal interpolating.

**Figure 7:**
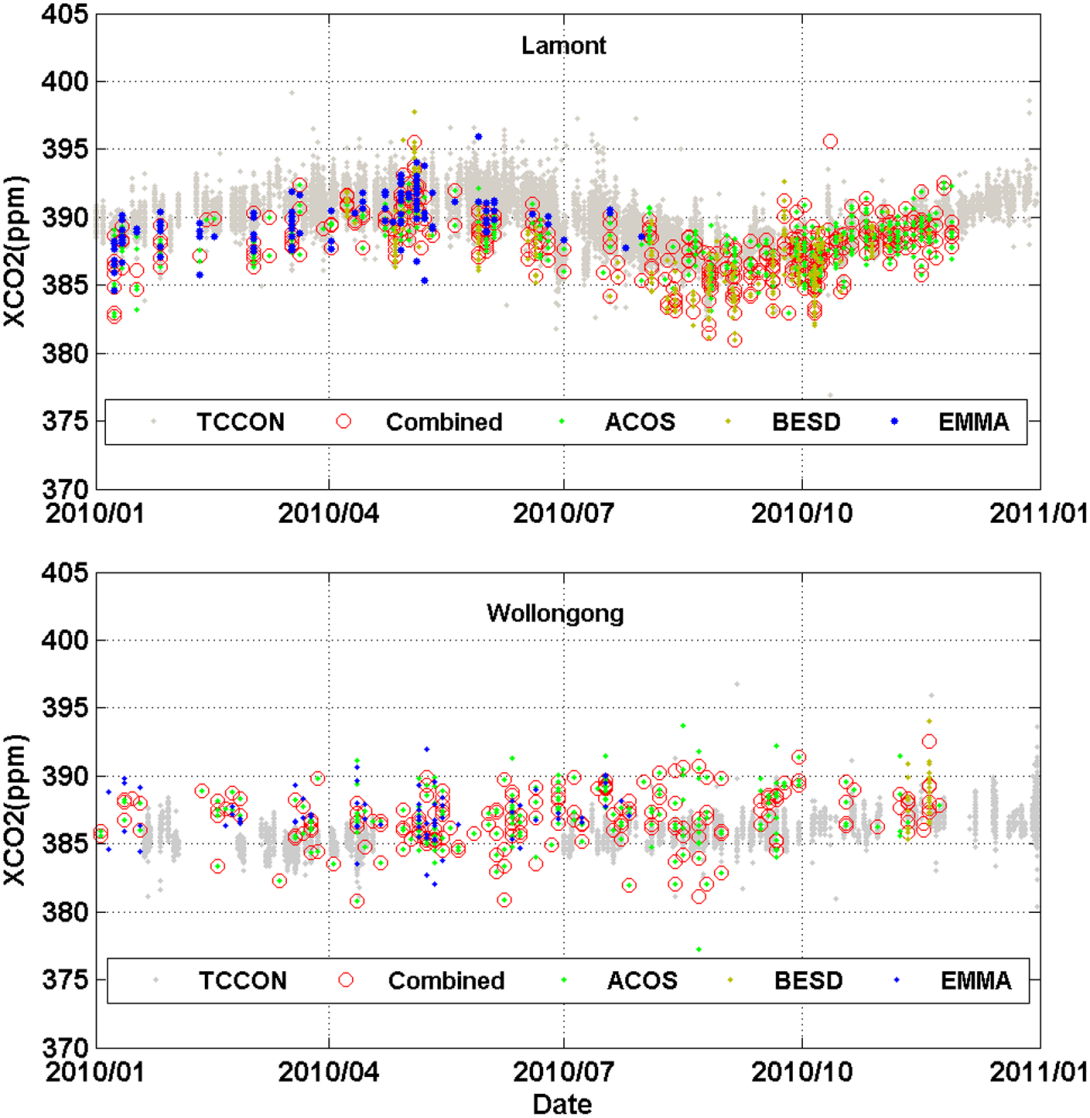
Comparison of XCO_2_ measurements from TCCON, ACOS, BESD, EMMA, and our new combination method over Wollongong and Lamont sites (distance<0.25 degree, temporal difference<1 hour).

For evaluating the performance of our combination strategy, the combined XCO_2_ values are compared with that retrieved from ACOS and BESD as well as XCO_2_ in the EMMA database at two TCCON sites (Fig. 7). The results reveal that the XCO_2_ values from the combination method show generally consistent variation in time with TCCON measurements except for a small overall bias (especially for the Lamont site). On the whole, the new combined XCO_2_ product shows good consistency with the EMMA data, and they are comparable in terms of CO_2_ magnitude, while the combined XCO_2_ are shown with a longer time period, which is in line with the satellite observations, and possess more data points even over the same period.

## Discussions and Conclusions

Despite the fact that space-based measurements can provide a unique opportunity to map atmospheric CO_2_ over large areas, the number of valid CO_2_ measurements from a single space-based instrument is generally limited for a certain day over a specific region due to the presence of clouds. In addition, although these Level-2 XCO_2_ retrievals themselves are very important for inversion modeling of surface carbon sources/sinks, further comprehensive analysis by investigating the spatiotemporal full-coverage XCO_2_ (Level 3) distribution is needed for interpreting their significant scientific merit [32]. While the limited satellite observations restrict the generation of Level-3 XCO_2_ maps with high spatial and temporal resolutions when only a single satellite-based XCO_2_ dataset is considered. This is our main motivation in this paper.

In this study, a strategy for combining SCIAMACHY and GOSAT CO_2_ measurements has been proposed by fully accounting for the CO_2_ global bias, differences in averaging kernels and overpass times, and the Level-2 retrieval errors of the CO_2_ measurements being used. The results indicated that the average global coverage of both ACOS and BESD is less than 0.5% on a daily scale, and less than 6% on a monthly scale. While spatial coverage of combined XCO_2_ can reach up to 0.65% and 8.42% on daily and monthly scales, respectively, the comparison analysis reveals that the combined XCO_2_ product is consistent with TCCON and EMMA in both temporal variation and magnitude except for a small bias when compared with the TCCON measurements. All these findings herein prove the effectiveness of the combination method in supporting generation global full-coverage XCO_2_ maps with higher temporal and spatial sampling by jointly using two space-based XCO_2_ datasets. Similar to the existing studies (e.g. [32]–[34]), although these combined XCO_2_ are not intended to be used in inverse modeling studies, they deliver a key complement for such research, and can be deemed as an independent dataset for comparison with model predictions. Similar to the existing study [31], an improved fusion approach (based on multiple XCO_2_ datasets) to create Level-2 XCO_2_ measurements that can be directly used for inverse modeling is also attempted and will be presented in another paper.

A last point that needs to be addressed is that although we employed CO_2_ data of GOSAT and SCIAMACHY in this study, the proposed strategies are not restricted to such data. As a general strategy, it can be refined and adapted to further combine other XCO_2_ products, such as OCO-2, CarbonSat, etc. in the future, and even to be applied to the fusion of other trace gases, such as O_3_, CH_4_.
