# Correction: Combining XCO_2_ Measurements Derived from SCIAMACHY and GOSAT for Potentially Generating Global CO_2_ Maps with High Spatiotemporal Resolution

**DOI:** 10.1371/journal.pone.0148152

**Published:** 2016-01-25

**Authors:** Tianxing Wang, Jiancheng Shi, Yingying Jing, Tianjie Zhao, Dabin Ji, Chuan Xiong

The following information is missing from the Acknowledgements section: TCCON data were obtained from the TCCON Data Archive, hosted by the Carbon Dioxide Information Analysis Center (CDIAC)—tccon.onrl.gov.
